# A systematic review of randomised controlled trials, non-randomised controlled trials and observational studies to ascertain the role of pollen-specific immunotherapy in improving clinical outcomes in pollen-food (ORAL ALLERGY) syndrome

**DOI:** 10.1186/1939-4551-8-S1-A94

**Published:** 2015-04-08

**Authors:** Priya Bowry

**Affiliations:** 1 The Allergy Clinic, Kenya

## Background

Pollen Food(Oral allergy) Syndrome (PFS) is a food allergy which is caused by cross-reactivity between inhalant pollen allergens and food allergens, causing mild to severe symptoms upon ingestion of the cross-reacting foods. PFS has a high and increasing prevalence in pollen-allergic patients. The role of pollen-specific immunotherapy in improving clinical outcomes for patients with PFS has not been established. To date, there are no systematic reviews which have been identified in the background research in this field. Aim of the review was to evaluate the evidence and determine whether pollen-specific immunotherapy clinically improves symptoms of PFS in pollen-allergic patients.

## Methods

This review was undertaken in accordance with the Cochrane Handbook for Systematic Reviews and PRISMA. Comprehensive literature search was conducted to identify suitable studies including randomised controlled trials (RCTs), observational studies and non-randomised trials (NRCTs) which investigated the role of a pollen-specific immunotherapy for a minimum period of 1 year in the clinical improvement of PFS in pollen-allergic patients. Quality assessments were made for each study using two instruments to ensure methodological rigour. Raw outcome data was extracted and analysed for each of the study designs. Sensitivity and heterogeneity analysis were performed.

## Results

11 trials (506 patients) met the inclusion criteria. 4 were RCTs (n=120), 2 were NRCTs (n=102) and 5 were observational before-and-after (immunotherapy) studies (n=156). A total of 378 patients were analysed. 290 had immunotherapy and 88 did not. 156 of those patients receiving immunotherapy served as their own controls in the observational studies in the data analysis. Quality assessments demonstrated that several studies had limitations and were not all of strong methodological rigour. Most studies were underpowered. This meta-analysis shows trends favouring immunotherapy. However, there was considerable heterogeneity in the RCTs. The RCTs showed an overall pooled odds ratio (OR) of 0.36 (95% confidence interval [CI] 0.07, 2.00) favouring immunotherapy. In the sensitivity analysis, the effect of removing the outlier study resulted in a statistically significant OR of 0.19 (95% CI 0.05, 0.74) and a reduction in heterogeneity. For NRCTs, the pooled OR was 0.01 (95% CI 0.00, 0.05) and for Observational studies, the OR was 0.02 (95% CI 0.01, 0.07), favouring immunotherapy.

## Conclusions

This meta-analysis demonstrates potentially favourable effects of pollen-specific immunotherapy in improving clinical outcomes for PFS patients. However, the inadequacies of the reported trials analysed limit the reliability of these findings. Further research is warranted on the basis of these results, using robust clinical studies.

